# Finding Nemo: hybrid assembly with Oxford Nanopore and Illumina reads greatly improves the clownfish (*Amphiprion ocellaris*) genome assembly

**DOI:** 10.1093/gigascience/gix137

**Published:** 2018-01-12

**Authors:** Mun Hua Tan, Christopher M Austin, Michael P Hammer, Yin Peng Lee, Laurence J Croft, Han Ming Gan

**Affiliations:** 1 Centre for Integrative Ecology, School of Life and Environmental Sciences, Deakin University, Geelong, Victoria 3220, Australia; 2 Genomics Facility, Tropical Medicine and Biology Platform, Monash University Malaysia, Jalan Lagoon Selatan, Bandar Sunway 47500, Petaling Jaya, Selangor, Malaysia; 3 School of Science, Monash University Malaysia, Jalan Lagoon Selatan, Bandar Sunway 47500, Petaling Jaya, Selangor, Malaysia; 4 Museum and Art Gallery of the Northern Territory, Darwin 0801, Australia; 5 Malaysian Genomics Resource Centre Berhad, Mid Valley City 59200, Kuala Lumpur, Malaysia

**Keywords:** clownfish, long reads, genome, transcriptome, hybrid assembly

## Abstract

**Background:**

Some of the most widely recognized coral reef fishes are clownfish or anemonefish, members of the family Pomacentridae (subfamily: Amphiprioninae). They are popular aquarium species due to their bright colours, adaptability to captivity, and fascinating behavior. Their breeding biology (sequential hermaphrodites) and symbiotic mutualism with sea anemones have attracted much scientific interest. Moreover, there are some curious geographic-based phenotypes that warrant investigation. Leveraging on the advancement in Nanopore long read technology, we report the first hybrid assembly of the clown anemonefish (*Amphiprion ocellaris*) genome utilizing Illumina and Nanopore reads, further demonstrating the substantial impact of modest long read sequencing data sets on improving genome assembly statistics.

**Results:**

We generated 43 Gb of short Illumina reads and 9 Gb of long Nanopore reads, representing approximate genome coverage of 54× and 11×, respectively, based on the range of estimated k-mer-predicted genome sizes of between 791 and 967 Mbp. The final assembled genome is contained in 6404 scaffolds with an accumulated length of 880 Mb (96.3% BUSCO-calculated genome completeness). Compared with the Illumina-only assembly, the hybrid approach generated 94% fewer scaffolds with an 18-fold increase in N_50_ length (401 kb) and increased the genome completeness by an additional 16%. A total of 27 240 high-quality protein-coding genes were predicted from the clown anemonefish, 26 211 (96%) of which were annotated functionally with information from either sequence homology or protein signature searches.

**Conclusions:**

We present the first genome of any anemonefish and demonstrate the value of low coverage (∼11×) long Nanopore read sequencing in improving both genome assembly contiguity and completeness. The near-complete assembly of the *A. ocellaris* genome will be an invaluable molecular resource for supporting a range of genetic, genomic, and phylogenetic studies specifically for clownfish and more generally for other related fish species of the family Pomacentridae.

## Data Description

**Figure 1:**
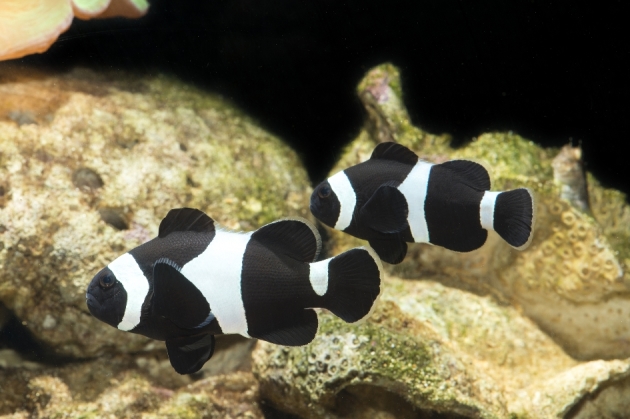
The clown anemonefish (*Amphiprion ocellaris*). Photo by Michael P. Hammer.

The clown anemonefish, *Amphiprion ocellaris* (Fig. 1, NCBI Taxon ID: 80 972, Fish Base ID:6509), is a well-known tropical marine fish species among the nonscientific community especially following the Pixar film *Finding Nemo* and its sequel *Finding Dory* [1]. The visual appeal of *A. ocellaris* due to its bright coloration and behaviour and ease of husbandry have maintained a strong global demand for this species in the marine aquarium trade, driving a fine balance between positive environmental awareness and sustainable ornamental use [1, 2]. Further, given high survival rates and ability to complete their life cycle in captivity, captive-breeding programs to partially sustain their global trade have been successful [3]. For the scientific community, *A. ocellaris* or anemonefishes in general are actively studied due to their intriguing reproductive strategy, i.e., sequential hermaphroditism [4–7] and mutualistic relationships with sea anemones [8–12]. Phenotypic body colour variation based on host-anemone use and geography also pose additional questions regarding adaptive genetic variation [13].

In recent years, concurrent with the advent of long read sequencing technologies [14], several studies have explored combining short but accurate Illumina reads with long but less accurate Nanopore/PacBio reads to obtain genome assemblies that are usually more contiguous with higher completeness than assemblies based on Illumina-only reads [15–19]. To further contribute to the evaluation of long read technology in fish genomics [15], we sequenced the whole genome of *A. ocellaris* using Oxford Nanopore and Illumina technologies and demonstrate that hybrid assembly of long and short reads greatly improved the quality of genome assembly.

## Whole-genome sequencing

Tissues for genome assembly and as reference material were sourced from the collection of the Museum and Art Gallery of the Northern Territory (NTM). The samples used for DNA extraction and subsequent whole-genome sequencing were from freshly vouchered captive bred *A. ocellaris* specimens, representing a unique black and white colour phenotype found only in the Darwin Harbour region, Australia (NTM A3764, A4496, A4497).

Genomic DNA was extracted from multiple fin clip and muscle samples using the E.Z.N.A. Tissue DNA Kit (Omega Bio-tek, Norcross, GA, USA). For Illumina library prep, approximately 1 μg of gDNA from isolate A3764 was sheared to 300 bp using a Covaris Focused-Ultrasonicator (Covaris, Woburn, MA, USA) and subsequently processed using the TruSeq DNA Sample Prep Kit (Illumina, San Diego, CA, USA) according to the manufacturer's instructions. Paired-end sequencing was performed on a single lane of HiSeq 2000 (Illumina, San Diego, CA, USA) located at the Malaysian Genomics Resource Centre Berhad. Two additional libraries were constructed from specimen NTM A3764, and both libraries were sequenced on the MiSeq (2 × 300 bp setting), located at the Monash University Malaysia Genomics Facility.

To generate Oxford Nanopore long reads, approximately 5 μg of gDNA was extracted from isolates NTM A4496 and A4497, size-selected (8–30 kb) with a BluePippin (Sage Science, Beverly, MA, USA), and processed using the Ligation Sequencing 1D Kit (Oxford Nanopore, Oxford, UK) according to the manufacturer's instructions. Three libraries were prepared and sequenced on 3 different R9.4 flowcells using the MinION portable DNA sequencer (Oxford Nanopore, Oxford, UK) for 48 hours.

## Sequence read processing

Raw Illumina short reads were adapter-trimmed with Trimmomatic v.0.36 (*ILLUMINACLIP:2:30:10, MINLEN:100*; Trimmomatic, RRID:SCR_011848) [20], followed by a screening for vectors and contaminants, using Kraken v.0.10.5 (Kraken, RRID:SCR_005484) [21] based on the MiniKraken DB. Kraken-unclassified reads, i.e., nonmicrobial/viral origin, were aligned to the complete mitogenome of NTM A3764 (see the Mitogenome Assembly section) to exclude sequences of organellar origin. This results in a total of 42.35 Gb of “clean” short reads. Nanopore reads were base-called from their raw FAST5 files using the Oxford Nanopore proprietary base-caller, Albacore, version 2.0.1. Applying a minimum length cutoff of 500 bp, this study produced a total of 8.95 Gbp in 895 672 Nanopore reads (N_50_: 12.7 kb). Sequencing statistics are available in Supplementary Table 1.

## Genome size estimation

K-mer counting with the “clean” Illumina reads was performed with Jellyfish v.2.2.6 (Jellyfish, RRID:SCR_005491) [22], generating k-mer frequency distributions of 17-, 21-, and 25-mers. These histograms were processed by GenomeScope [23], which estimated a genome size of 791 to 794 Mbp with approximately 80% of unique content and a heterozygosity level of 0.6% (Supplementary Fig. 1). Given that we had previously excluded adapters as well as sequences from contaminant or organellar sources, the max kmer coverage filter was not applied (*max kmer coverage: -1*). A separate estimation performed by BBMap [24] estimated a haploid genome size of 967 Mbp. The genome sizes estimated from both approaches are within the range of sizes listed for other *Amphiprion* species (792 Mb–1.2 Gb) as reported on the Animal Genome Size Database [25].

## Hybrid genome assembly

Short reads used for assemblies described in this study were only trimmed for adapters, but not for quality. Both short-read-only and hybrid *de novo* assemblies were performed with the Maryland Super-Read Celera Assembler v.3.2.2 (MaSuRCA, RRID:SCR_010691) [26]. During hybrid assembly, errors were encountered in the fragment correction step of the Celera Assembler (CA; Celera assembler, RRID:SCR_010750). To overcome this, given that the CA assembler is no longer maintained, we disabled the *frgcorr* step based on one of the developer's recommendations, and the hybrid assembly was subsequently improved with 10 iterations of Pilon v.1.22 (Pilon, RRID:SCR_014731) [27], using short reads to correct bases, fix misassemblies, and fill assembly gaps. To assess the completeness of the genome, Benchmarking Universal Single-Copy Orthologs v.3.0.2 (BUSCO, RRID:SCR_015008) [28] was used to locate the presence or absence of the Actinopterygii-specific set of 4584 single-copy orthologs (OrthoDB v9).

The short-read-only and hybrid assemblies yielded total assembly sizes of 851 Mb and 880 Mb, respectively. Statistics for assemblies for each Pilon iteration are available in Supplementary Table 2. Inclusion of Nanopore long reads for a hybrid assembly representing approximately ×11 genome coverage led to a 94% decrease in the number of scaffolds (>500 bp) from 106 526 to 6404 scaffolds and an 18-fold increase in the scaffold N_50_ length from 21 802 bp to 401 715 bp (Table 1). In addition, the genome completeness was also substantially improved in the hybrid assembly, with BUSCO detecting complete sequences of 96.3% (4417/4584) of single-copy orthologs in the Actinopterygii-specific dataset.

**Table 1:** Genome and transcriptome statistics of the clownfish (*Amphiprion ocellaris*) genome

	Illumina (≥500 bp)	Illumina + Nanopore (≥500 bp)
Genome assembly		
Contig statistics		
Number of contigs	133 997	7810
Total contig size, bp	851 389 851	880 159 068
Contig N_50_ size, bp	15 458	323 678
Longest contig, bp	204 209	2051 878
Scaffold statistics		
Number of scaffolds	106 526	6404
Total scaffold size, bp	852 602 726	880 704 246
Scaffold N_50_ size, bp	21 802	401 715
Longest scaffold, bp	227 111	3111 502
GC/AT/N, %	39.6/60.2/0.14	39.4/60.5/0.06
BUSCO genome completeness		
Complete	3691 (80.5%)	4417 (96.3%)
Complete and single copy	3600 (78.5%)	4269 (93.1%)
Complete and duplicated	91 (2.0%)	148 (3.2%)
Fragmented	534 (11.6%)	63 (1.4%)
Missing	359 (7.9%)	104 (2.3%)
Transcriptome assembly		
Number of contigs	25 364	
Total length, bp	68 405 796	
Contig N_50_ size, bp	3670	
BUSCO completeness		
Complete	4253 (92.8%)	
Complete and single-copy	4128 (90.1%)	
Complete and duplicated	125 (2.7%)	
Fragmented	127 (2.8%)	
Missing	204 (4.4%)	
Genome annotation		
Number of protein-coding genes	27 420	
Number of functionally annotated proteins	26 211	
Mean protein length	514 aa	
Longest protein	29 084 aa (titin protein)	
Average number (length) of exon per gene	9 (355 bp)	
Average number (length) of intron per gene	8 (1532 bp)	

## Transcriptome sequencing and assembly

Total RNA extraction from RNAshield-preserved whole-body and muscle tissues of isolate A4496 used Quick-RNA MicroPrep (Zymo Research Corpt, Irvine, CA, USA) according to the manufacturer's protocols. After assessing total RNA intactness on the Tapestation2100 (Agilent), mRNA was enriched using NEBNext Poly(A) mRNA Magnetic Isolation Kit (NEB, Ipwich, MA, USA) and processed with NEBNext Ultra RNA Library Prep Kit for Illumina (NEB, Ipwich, MA, USA). Libraries from both whole-body and muscle tissues were sequenced on a fraction of MiSeq V3 flowcell (1 × 150 bp). Single-end reads from both libraries in addition to 2 publicly available *A. ocellaris* transcriptome sequencing data (SRR5253145 and SRR5253146, Bioproject ID: PRJNA374650) were individually assembled using Scallop v0.10.2 [29] based on HiSat2 [30] alignment of RNA-sequencing reads to the newly generated *A. ocellaris* genome. The transcriptome assemblies were subsequently merged using the tr2aacds pipeline from the EvidentialGene [31] package and similarly assessed for completeness using BUSCO, version 3 [28]. The final nonredundant transcriptome assembly, which was subsequently used to annotate the *A. ocellaris* genome, contains 25 264 contigs/isotigs (putative transcripts) with an accumulated length of 68.4 Mb and BUSCO-calculated completeness of 92.8% (Table 1).

## Genome annotation

Protein-coding genes were predicted with the MAKER v.2.31.9 genome annotation pipeline (MAKER, RRID:SCR_005309) [32]. A total of 3 passes were run with MAKER2; the first pass was based on hints from the assembled transcripts as RNA-seq evidence (*est2genome*) and protein sequences from 11 fish species downloaded from Ensembl (Ensembl, RRID:SCR_002344) [33] (*protein2genome*), whereas the second and third passes included gene models trained from the first (and then second) passes with *ab initio* gene predictors SNAP (SNAP, RRID:SCR_002127) [34] and Augustus (Augustus: Gene Prediction, RRID:SCR_008417) [35]. In the final set of genes predicted, sequences with annotation edit distance (AED) values of less than 0.5 were retained. A small AED value suggests a lesser degree of difference between the predicted protein and the evidence used in the prediction (i.e., fish proteins, transcripts). This resulted in a final set of 27 240 protein-coding genes with an average AED of 0.14 (Table 1). A BUSCO analysis on the completeness of the predicted protein dataset detected the presence of 4259 (92.9%) single-copy orthologs from the Actinopterygii-specific dataset.

Further, to infer the putative function of these predicted proteins, NCBI’s *blastp* v.2.6.0 (*-evalue 1e-10, -seg yes, -soft_masking true, -lcase_masking*; BLASTP, RRID:SCR_001010) [36] was used to find homology to existing vertebrate sequences in the nonredundant (NR) database. Applying a hit fraction filter to include only hits with ≥70% target length fraction, the remaining unannotated sequences were subsequently aligned to all sequences in the NR database. With this method, 20 107 proteins (74%) were annotated with a putative function based on homology. Additionally, InterProScan v.5.26.65 (InterProScan, RRID:SCR_005829) [37] was used to examine protein domains, signatures, and motifs present in the predicted protein sequences. This analysis detected domains, signatures, or motifs for 26 211 proteins (96%). Overall, 96% of the predicted clownfish protein-coding genes were functionally annotated with information from at least 1 of the 2 approaches.

## Mitogenome recovery via genome skimming

Genome skimming [38, 39] was performed on 3 additional *A. ocellaris* individuals from known localities (Supplementary Table 3). Mitogenome assembly was performed with MITObim, version 1.9 (MITObim, RRID:SCR_015056) [40], using the complete mitogenome of *A. ocellaris* (GenBank: NC009065.1) as the bait for read mapping. The assembled mitogenomes were subsequently annotated with MitoAnnotator [41]. Consistent with the original broodstock collection from northern Australia, the captive-bred black and white *A. ocellaris* NTM A3764 exhibits strikingly high whole-mitogenome nucleotide identity (99.98%) to sample NTM A3708 as a wild collection from Darwin Harbour, Australia. In addition, the overall high pair-wise nucleotide identity (>98%) of NTM A3764 to newly generated and publicly available *A. ocellaris* whole mitogenomes further supports its morphological identification as *A. ocellaris* (Supplementary Table 3).

## Identification of the *cyp19a1a* gene associated with sexual differentiation

**Figure 2:**
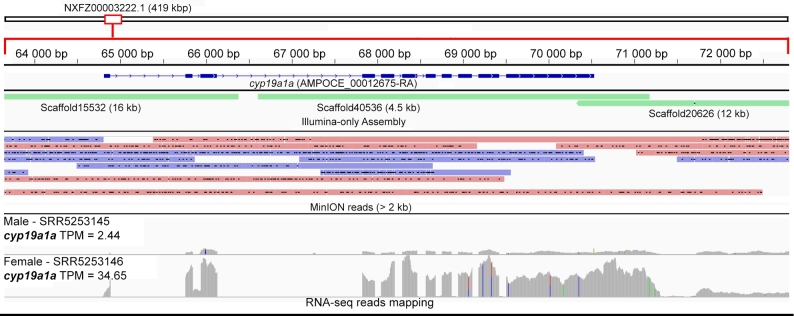
Mapping of MinION long reads, Illumina-assembled scaffolds, and RNA-sequencing reads of male and female *A. ocellaris* to the genomic region containing the *cyp19a1a* gene. Transcripts per million (TPM) values were calculated using Kallisto, version 0.43.1 [46].

The validated *cyp19a1a* enzyme of *Danio rerio* (Uniprot: O42145) was used as the query (E-value = 1e-10) for blastp search against the predicted *A. ocellaris* proteins. The top blast hit, AMPOCE_00 012675-RA (71.5% protein identity to O42145), was searched (tblastn) against the NCBI TSA database (Taxon: *Amphirion*) and showed strikingly high protein identity (99%) to a translated RNA transcript from *Amphiprion bicinctus* (c183337_g1_i2: GDCV01327693) [5]. The *cyp19a1a* gene codes for a steroidogenic enzyme that converts androgens into estrogens [42] were recently shown to be instrumental during sex change in *Amphiprion bicinctus*, as evidenced by significant correlation and differential expression of this gene between males and mature females [5]. We also observed a similar profile based on mapping of RNA reads from the publicly available male and female transcriptomes of *A. ocellaris* to the *cyp19a1a* gene region as visualized using the Integrative Genomics Viewer (Fig. 2) [43]. The *A. ocellaris cyp19a1a* gene is located on a 419-kb scaffold and is spanned by multiple Minimap2-aligned Nanopore reads [44]. It is noteworthy that in the Illumina-only assembly, this gene is fragmented and located on 3 relatively short scaffolds (Fig. 2).

## Conclusion

We present the first clownfish genome co-assembled with high-coverage Illumina short reads and low-coverage (∼11×) Nanopore long reads. Hybrid assembly of Illumina and Nanopore reads is one of the new features of the MaSuRCA assembler, version 3.2.2, which works by constructing long and accurate mega-reads from the combination of long and short read data. Although this is a relatively computationally intensive strategy with long run times, we observed substantial improvement in the genome statistics when compared with Illumina-only assembly. As Nanopore technology becomes more mature, it is likely that future de *novo* genome assembly will shift toward high-coverage long read–only assembly, followed by multiple iterations of genome polishing using Illumina reads.

## Availability of supporting data

Data supporting the results of this article are available in the *Giga*DB repository [45]. Raw Illumina and Nanopore reads generated in this study are available in the Sequence Read Archive (SRP123679), whereas the Whole Genome Shotgun project has been deposited at DDBJ/EMBL/GenBank under the accession NXFZ00000000, both under BioProject PRJNA407816.

## Abbreviations

bp: base pair; CDS: coding sequence; Gb: giga base; kb: kilo base; Mb: mega base; SRA: Sequence read archive; TE: transposable elements; TSA: transcriptome shotgun assembly.

## Additional files

Additional file 1: Figure S1: Genome profiling of *A. ocellaris* based on Illumina short reads.

Additional file 1: Table S1: Summary of raw reads generated from genome and transcriptome sequencing.

Additional file 1: Table S2: Assembly details after each pilon iteration.

Additional file 1: Table S3: Mitogenome similarity of *Amphiprion ocellaris* between the target sample (NTM A3764) and other isolates with known locality; body-colour phenotype is marked where known.

## Competing interests

The authors declare that they have no competing interests.

## Supplementary Material

GIGA-D-17-00310_Original_Submission.pdf

GIGA-D-17-00310_Revision_1.pdf

Response_to_Reviewer_Comments_Original_Submission.pdf

Reviewer_1_Report_(Original_Submission) -- Christiaan Henkel 04 Dec 2017 Reviewed

Reviewer_2_Report_(Original_Submission) -- Ole K Tørresen 06 Dec 2017 Reviewed

Supplemental material
