# Supplementary material for: Finding Nemo: hybrid assembly with Oxford Nanopore and Illumina reads greatly improves the clownfish (Amphiprion ocellaris) genome assembly
Source: Gigascience. 2018 Jan 12;7(3):gix137. doi: 10.1093/gigascience/gix137 (PMC5848817; doi:10.1093/gigascience/gix137)
Supplement: GIGA-D-17-00310_Original_Submission.pdf [file gix137_giga-d-17-00310_original_submission.pdf]

GigaScience

Finding Nemo 2.0: Hybrid assembly with Oxford Nanopore and Illumina reads dramatically improves the Clownfish (Amphiprion ocellaris) genome

--Manuscript Draft--

**Manuscript Number:**	GIGA-D-17-00310
**Full Title:**	Finding Nemo 2.0: Hybrid assembly with Oxford Nanopore and Illumina reads dramatically improves the Clownfish (Amphiprion ocellaris) genome
**Article Type:**	Data Note
**Funding Information:**	
**Abstract:**	Background: Some of the most widely recognised coral-reef fishes are clownfish or anemonefishes, members of the family Pomacentridae (subfamily: Amphiprioninae). They are popular aquarium species due to their bright colours, adaptability to captivity and fascinating behavior. Their breeding biology (sequential hermaphrodites) and symbiotic mutualism with sea anemones have attracted much scientific interest. Moreover there are some curious geographic-based phenotypes which warrant investigation. Leveraging on the advancement in Nanopore long read technology, we report the first hybrid assembly of the clown anemonefish (Amphiprion ocellaris) genome utilizing Illumina and Nanopore reads, further demonstrating the substantial impact of modest long read sequencing data sets on improving genome assembly statistics.<br><br>Findings: We generated 43 Gb of short Illumina reads and 9 Gb of long Nanopore reads representing an approximate genome coverage of 54× and 11×, respectively, based on an estimated k-mer predicted genome size of 794 Mb. The final assembled genome size is contained in 6,404 scaffolds with an accumulated length of 880 Mb (96.3% BUSCO-calculated genome completeness). Compared to the Illumina-only assembly, the hybrid approach generated 93% less scaffolds with 18-fold increase in N50 length (401 kb) and increased the genome completeness by an additional 16%. A total of 27,240 high quality protein-coding genes were predicted from the clown anemonefish, 26,211 (96%) of which were annotated functionally with information from either sequence homology or protein signature searches.<br><br>Conclusions: We present the first genome of any anemonefish and demonstrate the value of low coverage (~11×) long Nanopore reads sequencing in improving both genome contiguity and completeness. The near-complete genome of A. ocellaris will be an invaluable molecular resource for supporting a range of genetic, genomic and phylogenetic studies specifically for clownfish and more generally for other related fish species of the family Pomacentridae.
**Corresponding Author:**	Han Ming Gan<br><br>AUSTRALIA
**Corresponding Author Secondary Information:**	
**Corresponding Author's Institution:**	
**Corresponding Author's Secondary Institution:**	
**First Author:**	Mun Hua Tan
**First Author Secondary Information:**	
**Order of Authors:**	Mun Hua Tan
	Christopher Austin
	Michael Hammer
	Yin Peng Lee

	Laurence Croft
	Han Ming Gan
**Order of Authors Secondary Information:**	
**Opposed Reviewers:**	
**Additional Information:**	
**Question**	**Response**
Are you submitting this manuscript to a special series or article collection?	No
**Experimental design and statistics**<br><br>Full details of the experimental design and statistical methods used should be given in the Methods section, as detailed in our Minimum Standards Reporting Checklist. Information essential to interpreting the data presented should be made available in the figure legends.<br><br>Have you included all the information requested in your manuscript?	Yes
**Resources**<br><br>A description of all resources used, including antibodies, cell lines, animals and software tools, with enough information to allow them to be uniquely identified, should be included in the Methods section. Authors are strongly encouraged to cite Research Resource Identifiers (RRIDs) for antibodies, model organisms and tools, where possible.<br><br>Have you included the information requested as detailed in our Minimum Standards Reporting Checklist?	Yes
**Availability of data and materials**<br><br>All datasets and code on which the conclusions of the paper rely must be either included in your submission or deposited in publicly available repositories (where available and ethically appropriate), referencing such data using a unique identifier in the references and in the "Availability of Data and Materials" section of your manuscript.<br><br>Have you have met the above requirement as detailed in our Minimum	Yes

Standards Reporting Checklist?	

Powered by Editorial Manager® and ProduXion Manager® from Aries Systems Corporation

Manuscript

Click here to download Manuscript Nemo_GigaScience_151117.docx

Finding Nemo 2.0: Hybrid assembly with Oxford Nanopore and Illumina reads dramatically improves the Clownfish (*Amphiprion ocellaris*) genome

Mun Hua Tan[1,2,3#], Christopher M. Austin[1,2,3#], Michael P. Hammer[4], Yin Peng Lee[2,3], Laurence J. Croft[1,5], Han Ming Gan[1,2,3]*

[1] Centre for Integrative Ecology, School of Life and Environmental Sciences, Deakin University, Geelong, Victoria 3220, Australia

[2] Genomics Facility, Tropical Medicine and Biology Platform, Monash University Malaysia, Jalan Lagoon Selatan, Bandar Sunway 47500, Petaling Jaya, Selangor, Malaysia

[3] School of Science, Monash University Malaysia, Jalan Lagoon Selatan, Bandar Sunway 47500, Petaling Jaya, Selangor, Malaysia

[4] Museum and Art Gallery of the Northern Territory, Darwin 0801, Australia

[5] Malaysian Genomics Resource Centre Berhad, Boulevard Signature Office, Kuala Lumpur, Malaysia

[#] Equal contribution

*** Corresponding author:**

Name: Han Ming Gan, PhD

Address: Building Ka, Level 4, Room 4.338, Centre for Integrative Ecology, School of Life and Environmental Sciences, Deakin University, Waurn Ponds, Victoria 3216, Australia

Phone: (+61) 490786277

Email: han.gan@deakin.edu.au, ORCID: 0000-0001-7987-738X

Abstract

Background: Some of the most widely recognised coral-reef fishes are clownfish or anemonefishes, members of the family Pomacentridae (subfamily: Amphiprioninae). They are popular aquarium species due to their bright colours, adaptability to captivity and fascinating behavior. Their breeding biology (sequential hermaphrodites) and symbiotic mutualism with sea anemones have attracted much scientific interest. Moreover there are some curious geographic-based phenotypes which warrant investigation. Leveraging on the advancement in Nanopore long read technology, we report the first hybrid assembly of the clown anemonefish (*Amphiprion ocellaris*) genome utilizing Illumina and Nanopore reads, further demonstrating the substantial impact of modest long read sequencing data sets on improving genome assembly statistics.

Findings: We generated 43 Gb of short Illumina reads and 9 Gb of long Nanopore reads representing an approximate genome coverage of 54× and 11×, respectively, based on an estimated k-mer predicted genome size of 794 Mb. The final assembled genome size is contained in 6,404 scaffolds with an accumulated length of 880 Mb (96.3% BUSCO-calculated genome completeness). Compared to the Illumina-only assembly, the hybrid approach generated 93% less scaffolds with 18-fold increase in N_{50} length (401 kb) and increased the genome completeness by an additional 16%. A total of 27,240 high quality protein-coding genes were predicted from the clown anemonefish, 26,211 (96%) of which were annotated functionally with information from either sequence homology or protein signature searches.

Conclusions: We present the first genome of any anemonefish and demonstrate the value of low coverage (~11×) long Nanopore reads sequencing in improving both genome contiguity and completeness. The near-complete genome of *A. ocellaris* will be an invaluable molecular resource for supporting a range of genetic, genomic and phylogenetic studies specifically for clownfish and more generally for other related fish species of the family Pomacentridae.

Keywords: clownfish, long reads, genome, transcriptome, hybrid assembly

Data description

The clown anemonefish, *Amphiprion ocellaris* (NCBI Taxon ID: 80972, Fish Base ID:6509), is a well-known tropical marine fish species among the non-scientific community especially following the Pixar film "Finding Nemo" and its sequel "Finding Dory" [1]. The visual appeal of *A. ocellaris* due to its bright coloration and behaviour and ease of husbandry has maintained a strong global demand for this species in the marine aquarium trade driving a fine balance between positive environmental awareness versus sustainable ornamental use [1, 2]. Further, given high survival rates and ability to complete their lifecycle in captivity, captive-breeding programs to partially sustain their global trade have been successful [3]. For the scientific community, *A. ocellaris* or anemonefishes in general, are actively studied due to their intriguing reproductive strategy i.e. sequential hermaphroditism [4-7] and mutualistic relationships with sea anemones [8-12]. Phenotypic body-colour variation based on host-anemone use and geography also pose additional questions regarding adaptive genetic variation [13]

In recent years, concurrent with the advent of long read sequencing technologies [14], several studies have explored combining short but accurate Illumina reads with long but less accurate Nanopore/PacBio reads to obtain genome assemblies that are usually more contiguous with higher completeness than assemblies based on Illumina-only reads [15-19]. To further contribute to the evaluation of long read technology in fish genomics [15], we sequenced the whole genome of *A. ocellaris* using Oxford Nanopore and Illumina technologies and demonstrate that hybrid assembly of long and short reads greatly improved the quality of genome assembly.

Whole genome sequencing

Tissues for genome assembly and as reference material were sourced from the collection of the Museum and Art Gallery of the Northern Territory (NTM). The samples used for DNA extraction and subsequent whole genome sequencing were from freshly-vouchered captive bred *A. ocellaris* specimens representing a unique black and white colour phenotype found only in the Darwin Harbour region, Australia (NTM A3764, A4496, A4497).

Genomic DNA was extracted from multiple fin clip and muscle samples using E.Z.N.A.® Tissue DNA Kit (Omega Bio-tek, Norcross, GA). For Illumina library

prep, approximately 1µg of gDNA from isolate A3764 was sheared to 300 bp using a Covaris Focused-ultrasonicator (Covaris, Woburn, MA) and subsequently processed using TruSeq DNA sample prep kit (Illumina, San Diego, CA) according to the manufacturer's instructions. Paired-end sequencing was performed on a single lane of HiSeq 2000 (Illumina, San Diego, CA) located at the Malaysian Genomics Resource Centre Berhad. Two additional libraries were constructed from specimen NTM A3764 and both libraries were sequenced on the MiSeq (2×300 bp setting) located at the Monash University Malaysia Genomics Facility.

To generate Oxford Nanopore long reads, approximately 5 µg of gDNA was extracted from isolates NTM A4496 and A4497, size-selected (8 – 30 kb) with a BluePippin (Sage Science, Beverly, MA) and processed using the Ligation sequencing 1D kit (Oxford Nanopore, UK) according to the manufacturer's instructions. Three libraries were prepared and sequenced on three different R9.4 flowcells using the MinION portable DNA sequencer (Oxford Nanopore, UK) for 48 hours.

Sequence read processing

Raw Illumina short reads were adapter-trimmed with Trimmomatic v.0.36 (*ILLUMINACLIP:2:30:10, MINLEN:100*) [20] followed by a screening for vectors and contaminants, using Kraken v.0.10.5 [21] based on the MiniKraken DB. Kraken-unclassified reads i.e. non-microbial/viral origin were aligned to the complete mitogenome of NTM A3764 (See "Mitogenome Assembly") to exclude sequences of organellar origin. This results in a total of 42.35 Gb "clean" short reads. Nanopore reads were base-called from their raw FAST5 files using the Oxford Nanopore propriety base-caller, Albacore version 2.0.1. Applying a minimum and maximum length cut-off of 500 bp and 1Mbp, respectively, this study produced a total of 8.95 Gbp in 895,672 Nanopore reads (N_{50}: 12.7 kb)

Genome size estimation

K-mer counting with the filtered Illumina reads was performed with Jellyfish v.2.2.6 [22], generating k-mer frequency distributions of 17-, 21- and 25-mers. These histograms were processed by GenomeScope [23] that estimated a genome size of 791 to 794 Mb with approximately 80% of unique content and a heterozygosity level of 0.6% (Supplemental Figure 1). Given that we had previously excluded adapters as

well as sequences from contaminant or organellar sources, the max kmer coverage filter was not applied (*max kmer coverage: -1*). The genome size estimated from this approach is within the range of sizes listed for other *Amphiprion* species (792 Mb - 1.2 Gb) as reported on the Animal Genome Size Database (http://www.genomesize.com accessed on 11th November 2017)

Hybrid genome assembly

Both short-read-only and hybrid *de novo* assemblies were performed with MaSuRCA v.3.2.2 [24]. During hybrid assembly, errors were encountered in the fragment correction step of the Celera Assembler (CA). To overcome this, given that the CA assembler is no longer maintained, we disabled the *frgcorr* step based on one of the developer's recommendations and the hybrid assembly was subsequently improved with 10 iterations of Pilon v.1.22 [25], using short reads to correct bases, fix mis-assemblies and fill assembly gaps. To assess the completeness of the genome, BUSCO v.3.0.2 [26] was used to locate the presence or absence of the Actinopterygii-specific set of 4,584 single copy orthologs (OrthoDB v9).

The short-read-only and hybrid assemblies yielded total assembly sizes of 851 Mb and 880Mb, respectively. Inclusion of Nanopore long reads for hybrid assembly representing approximately 11× genome coverage led to a 94% decrease in the number of scaffolds (> 500 bp) from 106,526 to 6,404 scaffolds and an 18-fold increase in the scaffold N_{50} length from 21,802 bp to 401,715 bp (Table 1). In addition, the genome completeness was also substantially improved in the hybrid assembly, with BUSCO detecting complete sequences of 96.3% (4,417/4,584) of single-copy orthologs in the Actinopterygii-specific dataset.

Transcriptome sequencing and assembly

Total RNA extraction from RNAshield-preserved whole body and muscle tissues of isolate A4496 used Quick-RNA MicroPrep (Zymo Research Corpt, Irvine, CA) according to the manufacturer's protocols. After assessing total RNA intactness on the Tapestation2100 (Agilent), mRNA was enriched using NEBNext Poly(A) mRNA magnetic isolation kit (NEB, Ipwich, MA) and processed with NEBNext Ultra RNA library prep kit for Illumina (NEB, Ipwich, MA). Libraries from both whole body and muscle tissues were sequenced on a fraction of MiSeq V3 flowcell (1×150 bp). Single-end reads from both libraries in addition to two publicly available *A. ocellaris*

transcriptome sequencing data (SRR5253145 and SRR5253146, Bioproject ID: PRJNA374650) were individually assembled using Scallop v0.10.2 [27] based on HiSat2 [28] alignment of RNA-sequencing reads to the newly generated *A. ocellaris* genome. The transcriptome assemblies were subsequently merged using the tr2aacds pipeline from the EvidentialGene [29] package and similarly assessed for completeness using BUSCO version 3 [26]. The final non-redundant transcriptome assembly, which was subsequently used to annotate the *A. ocellaris* genome, contains 25,264 contigs/isotigs (putative transcripts) with an accumulated length of 68.4 Mb and BUSCO-calculated completeness of 92.8% (Table 1).

Genome annotation

Protein-coding genes were predicted with the MAKER v.2.31.9 genome annotation pipeline [30]. A total of three passes were run with MAKER2; the first pass was based on hints from the assembled transcripts as RNA-seq evidence (*est2genome*) and protein sequences from 11 fish species downloaded from Ensembl [31] (*protein2genome*), whereas the second and third passes included gene models trained from the first (and then, second) passes with *ab initio* gene predictors SNAP [32] and Augustus [33]. In the final set of genes predicted, sequences with Annotation Edit Distance (AED) values less than 0.5 were retained. A small AED value suggests a lesser degree of difference between the predicted protein and the evidences used in the prediction (i.e. fish proteins, transcripts). This resulted in a final set of 27,240 protein-coding genes with an average AED of 0.14 (Table 1). A BUSCO analysis on the completeness of the predicted protein dataset detected the presence of 4,259 (92.9%) single-copy orthologs from the Actinopterygii-specific dataset.

Further, to infer putative function of these predicted proteins, NCBI's *blastp* v.2.6.0 (*-evalue 1e-10*, *-seg yes*, *-soft_masking true*, *-lcase_masking*) [34] was used to find homology to existing vertebrate sequences in the non-redundant (NR) database. Applying a hit fraction filter to include only hits with ≥70 % target length fraction, the remaining un-annotated sequences were subsequently aligned to all sequences in the NR database. With this method, 20,107 proteins (74%) were annotated with a putative function based on homology. Additionally, InterProScan v.5.26.65 [35] was used to examine protein domains, signatures and motifs present in the predicted protein sequences. This analysis detected domains, signatures or motifs for 26,211 proteins (96%). Overall, 96% of the predicted clown fish protein-coding genes were

functionally annotated with information from at least one of the two approaches.

Mitogenome recovery via genome skimming

Genome skimming [36, 37] was performed on three additional *A. ocellaris* individuals from known locality (Supplemental Table 1). Mitogenome assembly was performed with MITObim version 1.9 [38] using the complete mitogenome of *A. ocellaris* (GenBank: NC009065.1) as the bait for read mapping. The assembled mitogenomes were subsequently annotated with MitoAnnotator [39]. Consistent with original broodstock collection from northern Australia, the captive bred black and white *A. ocellaris* NTM A3764 exhibits strikingly high whole mitogenome nucleotide identity (99.98%) to sample NTM A3708 as a wild collection from Darwin Harbour, Australia. In addition, the overall high pair-wise nucleotide identity (> 98%) of NTM A3764 to newly generated and publicly available *A. ocellaris* whole mitogenomes further supports its morphological identification as *A. ocellaris* (Supplemental Table 1).

Identification of the *cyp19a1a* gene associated with sexual differentiation

The validated *cyp19a1a* enzyme of *Danio rerio* (Uniprot: O42145) was used as the query (E-value = 1e-10) for blastp search against the predicted *A. ocellaris* proteins. The top blast hit, AMPOCE_00012675-RA (71.5% protein identity to O42145), was searched (tblastn) against the NCBI TSA database (Taxon: *Amphirion*) and showed strikingly high protein identity (99%) to a translated RNA transcript from *Amphiprion bicinctus* (c183337_g1_i2 : GDCV01327693) [5]. The *cyp19a1a* gene codes for a steroidogenic enzyme that converts androgens into estrogens [40] and was recently shown to be instrumental during sex change in *Amphiprion bicinctus* as evidenced by significant correlation and differential expression of this gene between male and mature females [5]. We also observed similar profile based on mapping of RNA reads from the publicly available male and female transcriptomes of *A. ocellaris* to the *cyp19a1a* gene region as visualized using Integrative Genomics Viewer [41] (Figure 2). The *A. ocellaris cyp19a1a* gene is located on a 419 kb scaffold and is spanned by multiple Minimap2-aligned Nanopore reads [42]. It is noteworthy that in the Illumina-only assembly, this gene is fragmented and located on 3 relatively short scaffolds (Figure 2).

Conclusion

We present the first clownfish genome and demonstrate the value of low coverage (~11×) Nanopore long read sequencing in improving both genome contiguity and completeness. The near-complete genome of *A. ocellaris* will be an invaluable molecular resource for supporting a range of genetic, genomic, and phylogenetic studies specifically for clownfish and more generally for other related fish species of the family Pomacentridae.

Availability of supporting data

Data supporting the results of this article will be available in the GigaDB repository. Raw Illumina and Nanopore reads generated in this study are available in the Sequence Read Archive (SRP123679) whereas Whole Genome Shotgun project has been deposited at DDBJ/EMBL/GenBank under the accession NXFZ00000000, both under BioProject PRJNA407816.

Acknowledgements

This study was funded by Monash University Malaysia Tropical and Biology Multidisciplinary Platform.

Competing interests

The authors declare that they have no competing interests

Figure Legends

Figure 1. The clown anemonefish (*Amphiprion ocellaris*): Photo by Michael P. Hammer

Figure 2. Mapping of MinION long reads, Illumina-assembled scaffolds and RNA-sequencing reads of male and female *A. ocellaris* to genomic region containing the *cyp19a1a* gene. Transcripts per million (TPM) values were calculated using Kallisto version 0.43.1 [43].

Supplemental Figure 1. Genome profiling of *A. ocellaris* based on Illumina short reads

References

1. Militz TA and Foale S. The “Nemo Effect”: Perception and reality of Finding Nemo's impact on marine aquarium fisheries. Fish and Fisheries. 2017;18 3:596-606.
2. Madduppa HH, von Juterzenka K, Syakir M and Kochzius M. Socio-economy of marine ornamental fishery and its impact on the population structure of the clown anemonefish Amphiprion ocellaris and its host anemones in Spermonde Archipelago, Indonesia. Ocean & Coastal Management. 2014;100 Supplement C:41-50. doi:https://doi.org/10.1016/j.ocecoaman.2014.07.013.
3. Hall H and Warmolts D. The Role of Public Aquariums in the Conservation and Sustainability of the Marine Ornamentals Trade. Marine Ornamental Species. Blackwell Publishing Company; 2008. p. 305-24.
4. Madhu R, Madhu K and Retheesh T. Life history pathways in false clown Amphiprion ocellaris Cuvier, 1830: A journey from egg to adult under captive condition. Journal of the Marine Biological Association of India. 2012;54 1:77-90.
5. Casas L, Saborido-Rey F, Ryu T, Michell C, Ravasi T and Irigoien X. Sex Change in Clownfish: Molecular Insights from Transcriptome Analysis. Scientific Reports. 2016;6:35461. doi:10.1038/srep35461.
6. Buston P. Social hierarchies: size and growth modification in clownfish. Nature. 2003;424 6945:145-6.
7. Kobayashi Y, Horiguchi R, Miura S and Nakamura M. Sex- and tissue-specific expression of P450 aromatase (cyp19a1a) in the yellowtail clownfish, Amphiprion clarkii. Comp Biochem Physiol A Mol Integr Physiol. 2010;155 2:237-44.
8. Davenport D and Norris KS. Observations on the symbiosis of the sea anemone Stoichactis and the pomacentrid fish, Amphiprion percula. The Biological Bulletin. 1958;115 3:397-410.
9. Arvedlund M and Nielsen LE. Do the anemonefish Amphiprion ocellaris (Pisces: Pomacentridae) imprint themselves to their host sea anemone Heteractis magnifica (Anthozoa: Actinidae)? Ethology. 1996;102 2:197-211.
10. Mariscal RN. An experimental analysis of the protection of Amphiprion xanthurus Cuvier & Valenciennes and some other anemone fishes from sea anemones. Journal of Experimental Marine Biology and Ecology. 1970;4 2:134-49.
11. Hattori A. Coexistence of two anemonefishes, Amphiprion clarkii and A. perideraion, which utilize the same host sea anemone. Environmental Biology of Fishes. 1995;42 4:345-53.
12. Schmiege PF, D’Aloia CC and Buston PM. Anemonefish personalities influence the strength of mutualistic interactions with host sea anemones. Marine Biology. 2017;164 1:24.
13. Allen GR. Damselfishes of the world. Melle, Germany: Mergus Publishers; 1991.
14. Heather JM and Chain B. The sequence of sequencers: The history of sequencing DNA. Genomics. 2016;107 1:1-8. doi:https://doi.org/10.1016/j.ygeno.2015.11.003.
15. Austin CM, Tan MH, Harrisson KA, Lee YP, Croft LJ, Sunnucks P, et al. De novo genome assembly and annotation of Australia's largest freshwater fish,

the Murray cod (Maccullochella peelii), from Illumina and Nanopore sequencing read. GigaScience. 2017;6 8:1-6. doi:10.1093/gigascience/gix063.

16. Gan HM, Lee YP and Austin CM. Nanopore Long-Read Guided Complete Genome Assembly of Hydrogenophaga intermedia, and Genomic Insights into 4-Aminobenzenesulfonate, p-Aminobenzoic Acid and Hydrogen Metabolism in the Genus Hydrogenophaga. Frontiers in Microbiology. 2017;8 1880 doi:10.3389/fmicb.2017.01880.

17. Zimin AV, Puiu D, Hall R, Kingan S, Clavijo BJ and Salzberg SL. The first near-complete assembly of the hexaploid bread wheat genome, Triticum aestivum. GigaScience. 2017:gix097-gix. doi:10.1093/gigascience/gix097.

18. Zimin AV, Stevens KA, Crepeau MW, Puiu D, Wegrzyn JL, Yorke JA, et al. An improved assembly of the loblolly pine mega-genome using long-read single-molecule sequencing. GigaScience. 2017;6 1:1-4. doi:10.1093/gigascience/giw016.

19. Zimin AV, Puiu D, Luo M-C, Zhu T, Koren S, Marcais G, et al. Hybrid assembly of the large and highly repetitive genome of Aegilops tauschii, a progenitor of bread wheat, with the mega-reads algorithm. Genome Research. 2017; doi:10.1101/gr.213405.116.

20. Bolger AM, Lohse M and Usadel B. Trimmomatic: a flexible trimmer for Illumina sequence data. Bioinformatics. 2014;30 15:2114-20.

21. Wood DE and Salzberg SL. Kraken: ultrafast metagenomic sequence classification using exact alignments. Genome biology. 2014;15 3:R46.

22. Marçais G and Kingsford C. A fast, lock-free approach for efficient parallel counting of occurrences of k-mers. Bioinformatics. 2011;27 6:764-70. doi:10.1093/bioinformatics/btr011.

23. Vurture GW, Sedlazeck FJ, Nattestad M, Underwood CJ, Fang H, Gurtowski J, et al. GenomeScope: fast reference-free genome profiling from short reads. Bioinformatics. 2017;33 14:2202-4. doi:10.1093/bioinformatics/btx153.

24. Zimin AV, Marçais G, Puiu D, Roberts M, Salzberg SL and Yorke JA. The MaSuRCA genome assembler. Bioinformatics. 2013;29 21:2669-77.

25. Walker BJ, Abeel T, Shea T, Priest M, Abouelliel A, Sakthikumar S, et al. Pilon: an integrated tool for comprehensive microbial variant detection and genome assembly improvement. PloS one. 2014;9 11:e112963.

26. Simão FA, Waterhouse RM, Ioannidis P, Kriventseva EV and Zdobnov EM. BUSCO: assessing genome assembly and annotation completeness with single-copy orthologs. Bioinformatics. 2015;31 19:3210-2.

27. Shao M and Kingsford C. Accurate assembly of transcripts through phase-preserving graph decomposition. Nature Biotechnology. 2017; doi:10.1038/nbt.4020 https://www.nature.com/articles/nbt.4020#supplementary-information.

28. Kim D, Langmead B and Salzberg SL. HISAT: a fast spliced aligner with low memory requirements. Nature Methods. 2015;12:357. doi:10.1038/nmeth.3317 https://www.nature.com/articles/nmeth.3317#supplementary-information.

29. Gilber D. Gene-omes built from mRNA seq not genome DNA. F1000Research. 2016;5 1695:1. doi:10.7490/f1000research.1112594.1.

30. Holt C and Yandell M. MAKER2: an annotation pipeline and genome-database management tool for second-generation genome projects. BMC bioinformatics. 2011;12 1:491.

31. Hubbard T, Barker D, Birney E, Cameron G, Chen Y, Clark L, et al. The Ensembl genome database project. Nucleic acids research. 2002;30 1:38-41.
32. Korf I. Gene finding in novel genomes. BMC bioinformatics. 2004;5 1:59.
33. Stanke M, Steinkamp R, Waack S and Morgenstern B. AUGUSTUS: a web server for gene finding in eukaryotes. Nucleic acids research. 2004;32 suppl_2:W309-W12.
34. Boratyn GM, Camacho C, Cooper PS, Coulouris G, Fong A, Ma N, et al. BLAST: a more efficient report with usability improvements. Nucleic acids research. 2013;41 W1:W29-W33.
35. Zdobnov EM and Apweiler R. InterProScan–an integration platform for the signature-recognition methods in InterPro. Bioinformatics. 2001;17 9:847-8.
36. Gan HM, Schultz MB and Austin CM. Integrated shotgun sequencing and bioinformatics pipeline allows ultra-fast mitogenome recovery and confirms substantial gene rearrangements in Australian freshwater crayfishes. BMC evolutionary biology. 2014;14 1:19.
37. Grandjean F, Tan MH, Gan HM, Lee YP, Kawai T, Distefano RJ, et al. Rapid recovery of nuclear and mitochondrial genes by genome skimming from Northern Hemisphere freshwater crayfish. Zoologica Scripta. 2017.
38. Hahn C, Bachmann L and Chevreux B. Reconstructing mitochondrial genomes directly from genomic next-generation sequencing reads—a baiting and iterative mapping approach. Nucleic acids research. 2013;41 13:e129-e.
39. Iwasaki W, Fukunaga T, Isagozawa R, Yamada K, Maeda Y, Satoh TP, et al. MitoFish and MitoAnnotator: A mitochondrial genome database of fish with an accurate and automatic annotation pipeline. Molecular biology and evolution. 2013;30 11:2531-40.
40. Kallivretaki E, Eggen R, Neuhauss S, Alberti M, Kausch U and Segner H. Aromatase in zebrafish: a potential target for endocrine disrupting chemicals. Mar Environ Res. 2006;62 90:7.
41. Thorvaldsdóttir H, Robinson JT and Mesirov JP. Integrative Genomics Viewer (IGV): high-performance genomics data visualization and exploration. Briefings in bioinformatics. 2013;14 2:178-92.
42. Li H. Minimap2: fast pairwise alignment for long nucletide sequences. arXiv. 2017;1708:Artciel 01492.
43. Bray NL, Pimentel H, Melsted P and Pachter L. Near-optimal probabilistic RNA-seq quantification. Nat Biotech. 2016;34 5:525-7. doi:10.1038/nbt.3519 http://www.nature.com/nbt/journal/v34/n5/abs/nbt.3519.html#supplementary-information.

Table 1. Genome and Transcriptome statistics of the clownfish (*Amphiprion ocellaris*) genome

	**Illumina (≥500bp)**	**Illumina + Nanopore (≥500bp)**
**Genome Assembly**		
Contig statistics		
Number of contigs	133,997	7,810
Total contig size (bp)	851,389,851	880,159,068
Contig N_{50} size (bp)	15,458	323,678
Longest contig (bp)	204,209	2,051,878
Scaffold statistics		
Number of scaffolds	106,526	6,404
Total scaffold size (bp)	852,602,726	880,704,246
Scaffold N_{50} size (bp)	21,802	401,715
Longest scaffold (bp)	227,111	3,111,502
GC / AT / N (%)	39.6 / 60.2 / 0.14	39.4 / 60.5 / 0.06
BUSCO Genome Completeness		
Complete	3,691 (80.5%)	4,417 (96.3%)
Complete and single copy	3,600 (78.5%)	4,269 (93.1%)
Complete and duplicated	91 (2.0%)	148 (3.2%)
Fragmented	534 (11.6%)	63 (1.4%)
Missing	359 (7.9%)	104 (2.3%)
**Transcriptome Assembly**		
Number of contigs	25,364	
Total length (bp)	68,405,796	
Contig N_{50} size (bp)	3,670	
BUSCO completeness		
Complete	4,253 (92.8%)	
Complete and single-copy	4,128 (90.1%)	
Complete and duplicated	125 (2.7%)	
Fragmented	127 (2.8%)	
Missing	204 (4.4%)	
**Genome Annotation**		
Number of protein-coding genes	27,420	
Number of functionally-annotated proteins	26,211	

Mean protein length	514 aa
Longest protein	29,084 aa (titin protein)
Average number (length) of exon per gene	9 (355 bp)
Average number (length) of intron per gene	8 (1,532 bp)

Figure 1

Click here to download Figure Figure1.jpg

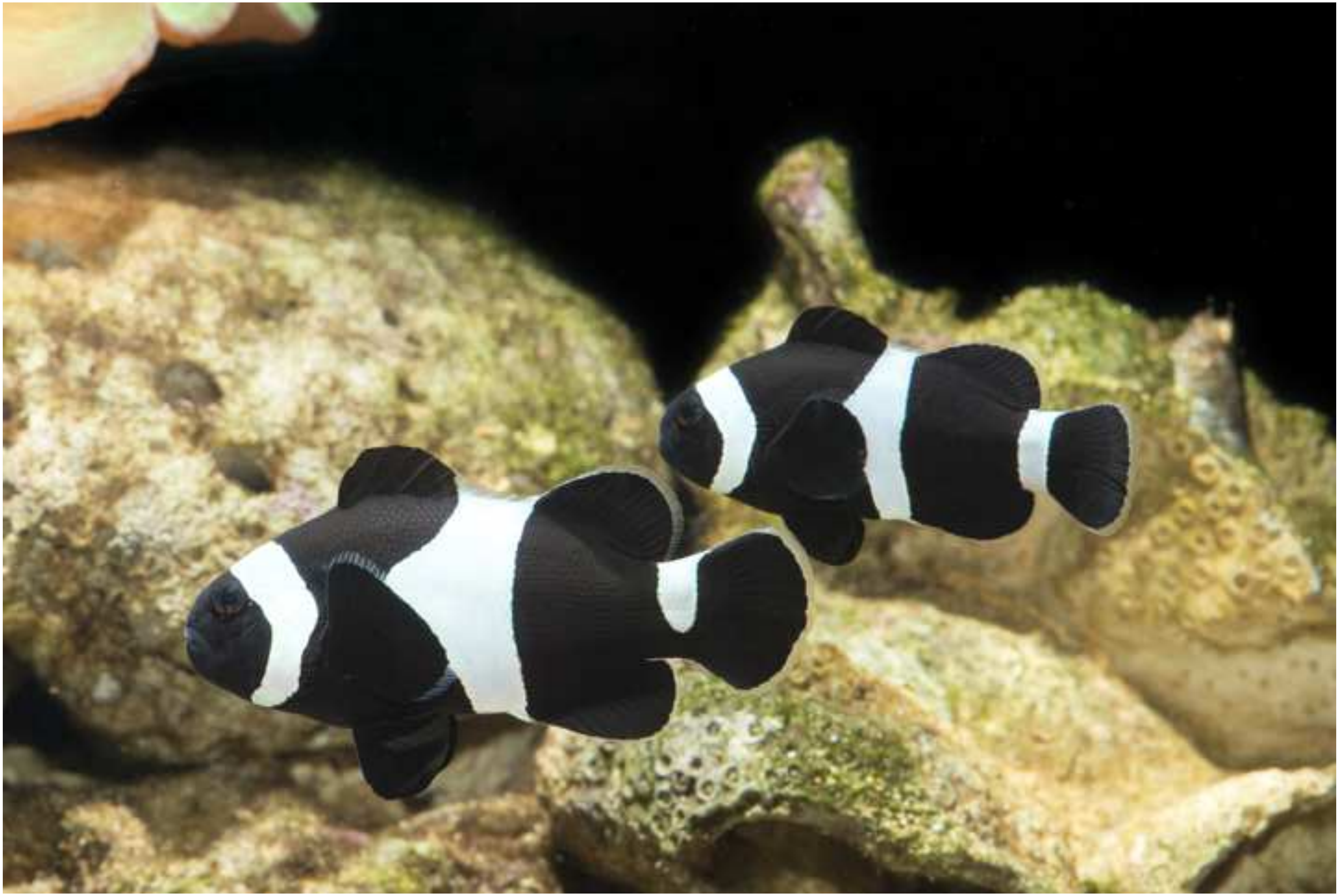

Figure 2

Click here to download Figure Figure2.tif

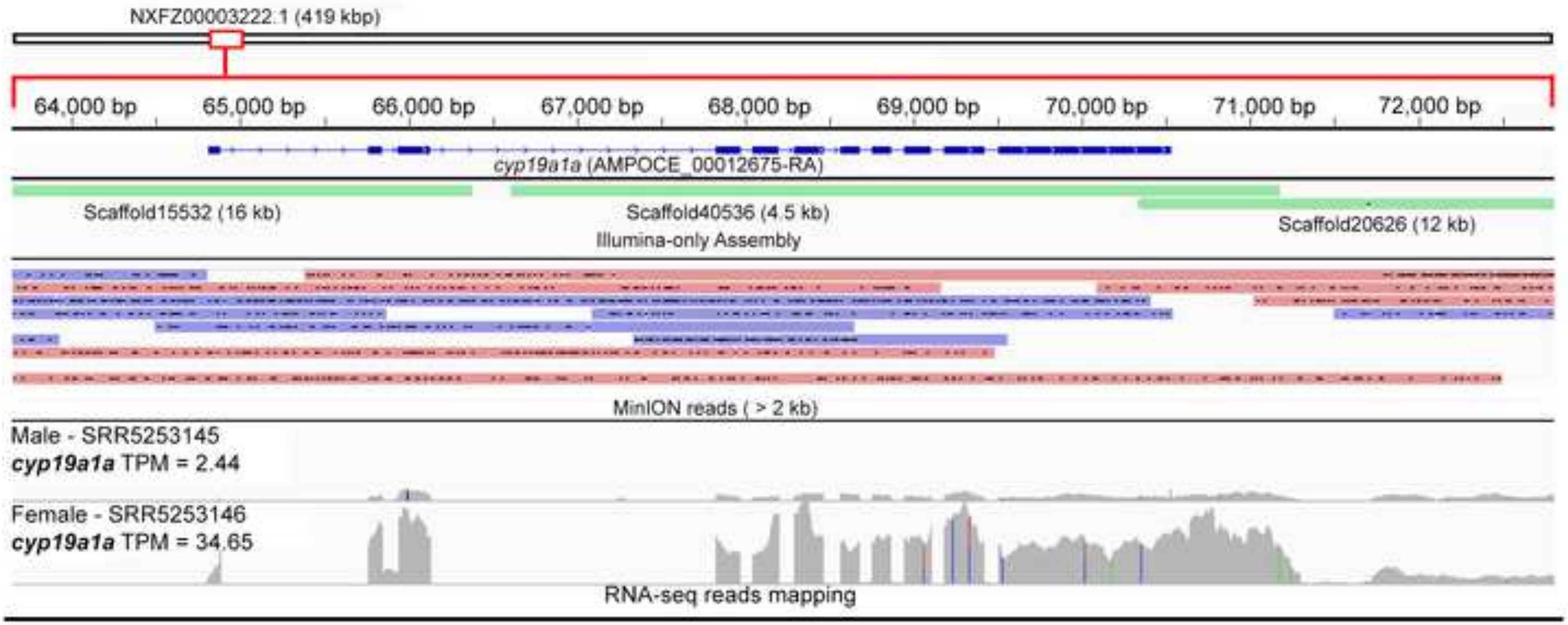

Supplementary Figure 1

Click here to access/download
Supplementary Material
SupplementalFigure1.tif

Supplementary Table 1

Click here to access/download
Supplementary Material
Supplemental Table 1_151117.docx
