# Supplementary material for: Finding Nemo: hybrid assembly with Oxford Nanopore and Illumina reads greatly improves the clownfish (Amphiprion ocellaris) genome assembly
Source: Gigascience. 2018 Jan 12;7(3):gix137. doi: 10.1093/gigascience/gix137 (PMC5848817; doi:10.1093/gigascience/gix137)
Supplement: Supplemental material [file gix137_supp.zip › Supplemental Table 1_091217.docx]

Supplemental Table 1: Summary of raw reads generated from genome and transcriptome sequencing

|  | **Sample ID** | **Illumina HiSeq** | | **Illumina MiSeq** | | **Nanopore MinION** | |
| --- | --- | --- | --- | --- | --- | --- | --- |
|  |  | **# reads** | **# bases** | **# reads** | **# bases** | **# reads** | **# bases** |
| Genome | A3764 | 272,481,374 | 27,520,600000 | 51,555,756 | 15,679,200,786 | - | - |
|  | A4496 | - | - | - | - | 455,344 | 3,878,154,428 |
|  | A4497 | - | - | - | - | 380,567 | 3,842,230,476 |
| Transcriptome | A4496 (whole body) | - | - | 5,273,318 | 761,960,221 | - | - |
|  | A4496 (muscle) | - | - | 7,156,366 | 1,039,277,557 | - | - |
