# Supplementary material for: Finding Nemo: hybrid assembly with Oxford Nanopore and Illumina reads greatly improves the clownfish (Amphiprion ocellaris) genome assembly
Source: Gigascience. 2018 Jan 12;7(3):gix137. doi: 10.1093/gigascience/gix137 (PMC5848817; doi:10.1093/gigascience/gix137)
Supplement: Supplemental material [file gix137_supp.zip › Supplemental Table 2_091217.docx]

Supplemental Table 3: Assembly details after each pilon iteration

| Iteration # | Number of changes | N50 (bp) | Number of gaps | Number of N’s |
| --- | --- | --- | --- | --- |
| 1 | 426,087 | 401,651 | 1450 | 560,882 |
| 2 | 130,089 | 401,666 | 1433 | 555,408 |
| 3 | 69,112 | 401,710 | 1416 | 553,192 |
| 4 | 43,851 | 401,714 | 1414 | 550,779 |
| 5 | 30,948 | 401,714 | 1410 | 549,938 |
| 6 | 24,579 | 401,714 | 1408 | 545,769 |
| 7 | 20,526 | 401,713 | 1408 | 545,429 |
| 8 | 17,770 | 401,714 | 1407 | 545,284 |
| 9 | 15,842 | 401,717 | 1406 | 545,221 |
| 10 | 14,357 | 401,715 | 1406 | 545,178 |
