# Supplementary material for: Finding Nemo: hybrid assembly with Oxford Nanopore and Illumina reads greatly improves the clownfish (Amphiprion ocellaris) genome assembly
Source: Gigascience. 2018 Jan 12;7(3):gix137. doi: 10.1093/gigascience/gix137 (PMC5848817; doi:10.1093/gigascience/gix137)
Supplement: Supplemental material [file gix137_supp.zip › Supplemental Table 3_091217.docx]

Supplemental Table 1: Mitogenome similarity of *Amphiprion ocellaris* between the target sample (NTM A3764) and other isolates with known locality; body-colour phenotype is marked where known

| Accession Number/ ID | Location / isolation source | Country | Identity to  A3764(%) | Reference |
| --- | --- | --- | --- | --- |
| NTM A3764 | Captive bred (black & white) | Australia | 100.00 | This study |
| AB979697.1 | Chonburi | Thailand | 99.87 | Unpublished |
| NTM A3708 | Darwin Harbour, Northern Territory, Australia (black & white) | Australia | 99.87 | This study |
| NC_009065.1 | Unknown | Unknown | 99.56 | [1] |
| NTM A3722 | Arafura Sea, Northern Territory, Australia (orange & white) | Australia | 99.51 | This study |
| NTM A3045 | Seribu Islands, West Java (orange & white) | Indonesia | 98.97 | This study |
| AB980197.1 | Chonburi | Thailand | 98.80 | Unpublished |
|  |  |  |  |  |

1. Mabuchi K, Miya M, Azuma Y, Nishima M. 2007. Independent evolution of the specialized pharyngeal jaw apparatus in cichlid and labrid fishes. BMC Evol. Bio. 7:10.
