# Supplementary figures and images for: Finding Nemo: hybrid assembly with Oxford Nanopore and Illumina reads greatly improves the clownfish (Amphiprion ocellaris) genome assembly
Source: Gigascience. 2018 Jan 12;7(3):gix137. doi: 10.1093/gigascience/gix137 (PMC5848817; doi:10.1093/gigascience/gix137)

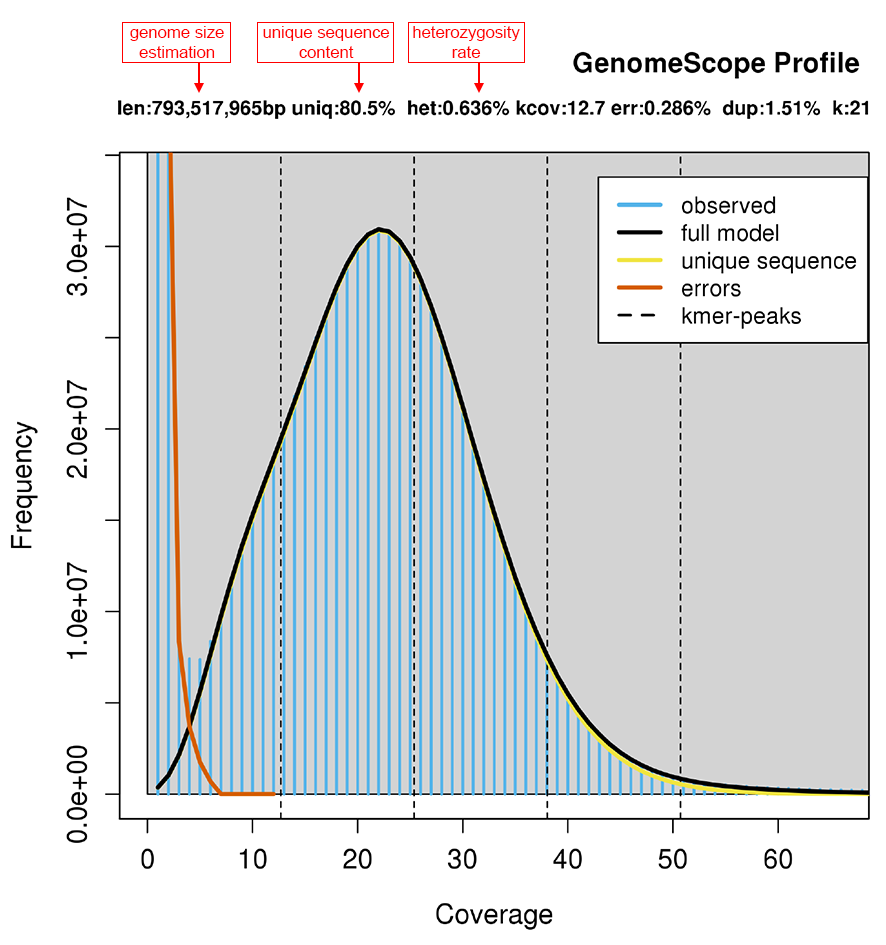

Supplement: Supplemental material [file gix137_supp.zip › Supplemental Figure 1_091217.tif]
