# Supplementary material for: Finding Nemo: hybrid assembly with Oxford Nanopore and Illumina reads greatly improves the clownfish (Amphiprion ocellaris) genome assembly
Source: Gigascience. 2018 Jan 12;7(3):gix137. doi: 10.1093/gigascience/gix137 (PMC5848817; doi:10.1093/gigascience/gix137)
Supplement: Response_to_Reviewer_Comments_Original_Submission.pdf [file gix137_response_to_reviewer_comments_original_submission.pdf]

Author's Response To Reviewer Comments

Reviewer #1
Comment 1:
In general, given the prominence (with 'dramatically') of nanopore data in the title, I would like to encourage the authors to elaborate on this aspect of the study in the Conclusion section. For example, why did you use this particular strategy (MaSuRCA assembler), and what are its strengths and weaknesses? How does long-read coverage affect the assembly process (this study uses only three nanopore flowcells - would this be a recommended efficient strategy to 'fix' any Illumina-based assembly)? How far are we from non-hybrid nanopore-based assemblies?

Response:
We've added discussion on this matter in the Conclusion. We chose MaSuRCA due to its demonstrated accuracy in addition to its ability to better utilize Nanopore reads in its initial assembly step. Despite producing a highly contiguous genome assembly of the clown fish, MaSuRCA hybrid assembly is an extremely computationally expensive and time-consuming. We are hopeful that the cost of Nanopore sequencing will reduce further especially with the recent release of PromethION which may revolutionize routine long read sequencing. However, as of now, high coverage long read sequencing is still financial challenging for smaller research group despite its indubitable value in assembling challenging (repetitive and/or heterozygous) genome. "Hybrid assembly of Illumina and Nanopore reads is one of the new features of the MaSuRCA assembler version 3.2.2 that works by constructing long and accurate mega-reads from the combination of long and short read data. Although this is a relatively computationally-intensive strategy with long run-times, we observed substantial improvement in the genome statistics when compared to Illumina-only assembly. As Nanopore long technology becomes more mature, it is likely that future de novo genome assembly will shift towards high coverage long read-only assembly followed by genome polishing using Illumina reads."

Comment 2:
Finally, a very similar genome project manuscript was recently posted on BioRxiv:
Anna Marcionetti et al., First draft genome assembly of an iconic clownfish species (Amphiprion frenatus), doi 10.1101/205443, 18 October 2017
The manuscripts do not cite each other, but arrive at similar genome assembly qualities using similar strategies.

Response:
Although the manuscript was posted on BioRxiv, the genome assembly itself is currently unavailable to the public and, according to the manuscript, will only be available in DRYAD repository (under 'Data Accessibility). So far, we were not able to locate the data on DRYAD.

Comment 3:
1. Line 128: an upper limit of 1 Mbp reads probably did not exclude anything. What was the actual longest read length?

Response:
We had initially obtained reads up to 40Mbp base-called from an older version of Albacore. But

after re-analysis with version 2.0.1 for this assembly, we had gotten far more sensible Nanopore read lengths and therefore did not apply any maximum length cutoff. We have removed the details on upper limit in the revised manuscript. The actual longest mappable read length for our Nanopore read dataset was 101,379bp, aligned to scaffold6249 (276,565bp) and contains genes with IDs: AMPOCE_00020294 and AMPOCE_00020295.

\---

Comment 4:

2. Line 131…/Supplemental Figure 1. Not all Illumina data were apparently used for the k-mer profile. Does this perhaps explain the considerable difference in estimated genome size and assembled genome size? If not, is there another explanation? Also, the legend to the figure ('genome profiling') could be more informative (e.g. genome size estimate…)

Response:

Based on this reviewer comment, we re-ran GenomeScope using the k-mer profile from all Illumina reads, which estimated genome sizes of 806 to 812 Mbp with different k-mer sizes, not too different from the initial number. A separate independent analysis was performed with BBMap, which estimated a haploid genome size of 967Mb. Given this result, the assembled genome size is well within the range of genome size estimated based on different methods. We have added the results from the BBMap analysis in the manuscript at lines 139 to 140. Supplemental Figure 1 has also been improved.

\---

Comment 5:

3. A (supplementary) table with sequencing statistics (yield for each type of data, incl. RNA-seq) would be appropriate.

Response:

Sequencing statistics has been summarized for each sample ID in the new Supplemental Table 1.

\---

Reviewer #2

Comment 1:

Title:

The reference to Nemo 2.0 and the phrase "dramatically improves" led me to believe that this was the second version of an already existing genome assembly. However, I could not find any other Amphiprion ocellaris assemblies by googling, besides a bioRxiv preprint of Amphiprion frenatus (https://www.biorxiv.org/content/early/2017/10/18/205443). I am not sure if this warrants changing the title, but please be aware of it. Also, I dislike using "genome" to refer to the genome assembly. I don't think you actually improve the genome present in the species.

Response:

The “2.0” in the title was included due to this manuscript (10.1016/j.gene.2006.03.028) with a similar title “Finding Nemo” and was not due to the recent A. frenatus genome in bioRxiv (see also Response to Reviewer’s 1 Comment 2). However, given that our study is not a follow-up of the phylogenetic study reported by Santini and Polacco (2006), we agree that this can be confusing to readers and have removed “2.0” from the title. We have also added “assembly” into the title as per reviewer’s suggestion and slightly modified the title.

\---

Comment 2:
Abstract:
Line 54: "93 % less scaffolds". This should be "fewer" if I'm not mistaken.

Response:
Replaced “less” with “fewer”.

Comment 3:
Lines 60-65: I prefer to see "genome assembly" instead of just "genome". I find it more accurately descriptive.

Response:
Edited title as per suggestion.

Comment 4:
Lines 120-125: The MaSuRCA quick start guide (ftp://ftp.genome.umd.edu/pub/MaSuRCA/MaSuRCA_QuickStartGuide.pdf) explicitly says that Illumina reads should not be pre-processed before providing them to MaSuRCA. It is not clear whether or not the "clean" reads were used in the assemblies. Were the "clean" reads used? Or were they only used for genome size estimation?

Response:
Since the raw reads obtained from the Illumina MiSeq already had adapter sequences trimmed off by default, reads used as input to MaSuRCA were only adapter-trimmed. However, no quality-trim/cleaning/error correction was performed on these reads since the program performs its own error correction steps. “Clean” reads which excluded bacteria/virus contaminants and mitochondrial origins were used mainly for genome size estimation to not underestimate the genome size when max kmer coverage is applied in GenomeScope.
“Kraken-unclassified reads i.e. non-microbial/viral origin were aligned to the complete mitogenome of NTM A3764 (See “Mitogenome Assembly”) to exclude sequences of organellar origin. This results in a total of 42.35 Gb “clean” short reads.”

Comment 5:
Line 149: "10 iterations of Pilon". Did you actually see any improvements after this many iterations? How did you assess the improvements?

Response:
We have added details on the assemblies obtained after each pilon iteration as Supplemental Table 3. While the contiguity of the assembly (indicated by N50) is not improved much after each iteration, the number of gaps and ‘N’s are reduced and these numbers are observed to plateau at later iterations (i9 or i10). In addition, pilon reports the changes made in the assembly. Based on this output, the number of changes made decreases with each iteration and eventually almost plateaus as well (Supplemental Table 3).

Comment 6:
Line 155: Here you claim a "94 % decrease in the number of scaffolds", while you claim 93 % in

the abstract. Which is correct? I guess both if you use different criteria for which scaffolds are included or not (>500 bp).

Response:
94% decrease is correct - fixed this in abstract
