# Supplementary material for: Finding Nemo: hybrid assembly with Oxford Nanopore and Illumina reads greatly improves the clownfish (Amphiprion ocellaris) genome assembly
Source: Gigascience. 2018 Jan 12;7(3):gix137. doi: 10.1093/gigascience/gix137 (PMC5848817; doi:10.1093/gigascience/gix137)
Supplement: Supplementary file 4 [file gix137_reviewer_1_report_(original_submission).pdf]

Reviewer Report

Title: Finding Nemo: Hybrid assembly with Oxford Nanopore and Illumina reads greatly improves the Clownfish (Amphiprion ocellaris) genome assembly

Version: Original Submission **Date:** 04 Dec 2017

Reviewer name: Christiaan Henkel

Reviewer Comments to Author:

This study describes the genome assembly of the clown anemonefish using a hybrid strategy, employing both short and long (nanopore) reads. The long reads contribute towards scaffold contiguity, whereas the short reads provide high sequence identity. Based on annotation and BUSCO statistics, this strategy is quite successful, yielding a genome assembly of high quality. By todays multiple-megabase-scaffold standards, the assembly is still relatively fragmented, presumably due to the modest amount of long-read data.

Overall, this is a well-executed study that would make for a relevant and timely publication. I have only a few minor suggestions (see below). In general, given the prominence (with 'dramatically') of nanopore data in the title, I would like to encourage the authors to elaborate on this aspect of the study in the Conclusion section. For example, why did you use this particular strategy (MaSuRCA assembler), and what are its strengths and weaknesses? How does long-read coverage affect the assembly process (this study uses only three nanopore flowcells - would this be a recommended efficient strategy to 'fix' any Illumina-based assembly)? How far are we from non-hybrid nanopore-based assemblies?

Finally, a very similar genome project manuscript was recently posted on BioRxiv:
Anna Marcionetti et al., First draft genome assembly of an iconic clownfish species (Amphiprion frenatus), doi 10.1101/205443, 18 October 2017
The manuscripts do not cite each other, but arrive at similar genome assembly qualities using similar strategies.

Specific minor comments:
1. Line 128: an upper limit of 1 Mbp reads probably did not exclude anything. What was the actual longest read length?
2. Line 131…/Supplemental Figure 1. Not all Illumina data were apparently used for the k-mer profile. Does this perhaps explain the considerable difference in estimated genome size and assembled genome size? If not, is there another explanation? Also, the legend to the figure ('genome profiling') could be more informative (e.g. genome size estimate…)
3. A (supplementary) table with sequencing statistics (yield for each type of data, incl. RNA-seq) would be appropriate.

Level of Interest

Please indicate how interesting you found the manuscript: An article whose findings are important to those with closely related research interests

Quality of Written English

Please indicate the quality of language in the manuscript: Acceptable

Declaration of Competing Interests

Please complete a declaration of competing interests, considering the following questions:

- Have you in the past five years received reimbursements, fees, funding, or salary from an organisation that may in any way gain or lose financially from the publication of this manuscript, either now or in the future?
- Do you hold any stocks or shares in an organisation that may in any way gain or lose financially from the publication of this manuscript, either now or in the future?
- Do you hold or are you currently applying for any patents relating to the content of the manuscript?
- Have you received reimbursements, fees, funding, or salary from an organization that holds or has applied for patents relating to the content of the manuscript?
- Do you have any other financial competing interests?
- Do you have any non-financial competing interests in relation to this paper?

If you can answer no to all of the above, write 'I declare that I have no competing interests' below. If your reply is yes to any, please give details below.

I have received travel reimbursements from Oxford Nanopore Technologies for speaking at several of their events.

I agree to the open peer review policy of the journal. I understand that my name will be included on my report to the authors and, if the manuscript is accepted for publication, my named report including any attachments I upload will be posted on the website along with the authors' responses. I agree for my report to be made available under an Open Access Creative Commons CC-BY license (http://creativecommons.org/licenses/by/4.0/). I understand that any comments which I do not wish to be included in my named report can be included as confidential comments to the editors, which will not be published.

I agree to the open peer review policy of the journal

To further support our reviewers, we have joined with Publons, where you can gain additional credit to further highlight your hard work (see: https://publons.com/journal/530/gigascience). On publication of this paper, your review will be automatically added to Publons, you can then choose whether or not to claim your Publons credit. I understand this statement.

Yes
