# Supplementary material for: Finding Nemo: hybrid assembly with Oxford Nanopore and Illumina reads greatly improves the clownfish (Amphiprion ocellaris) genome assembly
Source: Gigascience. 2018 Jan 12;7(3):gix137. doi: 10.1093/gigascience/gix137 (PMC5848817; doi:10.1093/gigascience/gix137)
Supplement: Supplementary file 5 [file gix137_reviewer_2_report_(original_submission).pdf]

Reviewer Report

Title: Finding Nemo: Hybrid assembly with Oxford Nanopore and Illumina reads greatly improves the Clownfish (Amphiprion ocellaris) genome assembly

Version: Original Submission **Date:** 06 Dec 2017

Reviewer name: Ole K Tørresen

Reviewer Comments to Author:

Title: Finding Nemo 2.0: Hybrid assembly with Oxford Nanopore and Illumina reads dramatically improves the Clownfish (Amphiprion ocellaris) genome

General comments
Luckily for the larger research community, long reads as generated by the machines produced by PacBio and Oxford Nanopore are becoming more and more affordable. The authors here demonstrate an approach where using moderate coverage of Nanopore long-reads drastically improves the contiguity compared to a pure Illumina-based genome assembly.

The clownfish are an interesting species because they are sequential hermaphrodites, where the largest male in a breeding group changes sex to a female if the female disappears. This aspect of their biology is unfortunately not accurately depicted in "Finding Nemo".

The authors have conducted a straightforward and solid work in sequencing, assembling and annotating the clownfish genome, and I have just a few comments to the manuscript.

Specific comments

Title:
The reference to Nemo 2.0 and the phrase "dramatically improves" led me to believe that this was the second version of an already existing genome assembly. However, I could not find any other Amphiprion ocellaris assemblies by googling, besides a bioRxiv preprint of Amphiprion frenatus (https://www.biorxiv.org/content/early/2017/10/18/205443). I am not sure if this warrants changing the title, but please be aware of it. Also, I dislike using "genome" to refer to the genome assembly. I don't think you actually improve the genome present in the species.

Abstract:
Line 54: "93 % less scaffolds". This should be "fewer" if I'm not mistaken.

Lines 60-65: I prefer to see "genome assembly" instead of just "genome". I find it more accurately descriptive.

Lines 120-125: The MaSuRCA quick start guide (ftp://ftp.genome.umd.edu/pub/MaSuRCA/MaSuRCA_QuickStartGuide.pdf) explicitly says that Illumina reads should not be pre-processed before providing them to MaSuRCA. It is not clear whether or not the "clean" reads were used in the assemblies. Were the "clean" reads used? Or were they only used for genome size estimation?

Line 149: "10 iterations of Pilon". Did you actually see any improvements after this many iterations? How did you assess the improvements?

Line 155: Here you claim a "94 % decrease in the number of scaffolds", while you claim 93 % in the abstract. Which is correct? I guess both if you use different criteria for which scaffolds are included or not (>500 bp).

Level of Interest

Please indicate how interesting you found the manuscript: An article of importance in its field

Quality of Written English

Please indicate the quality of language in the manuscript: Acceptable

Declaration of Competing Interests

Please complete a declaration of competing interests, considering the following questions:

- Have you in the past five years received reimbursements, fees, funding, or salary from an organisation that may in any way gain or lose financially from the publication of this manuscript, either now or in the future?
- Do you hold any stocks or shares in an organisation that may in any way gain or lose financially from the publication of this manuscript, either now or in the future?
- Do you hold or are you currently applying for any patents relating to the content of the manuscript?
- Have you received reimbursements, fees, funding, or salary from an organization that holds or has applied for patents relating to the content of the manuscript?
- Do you have any other financial competing interests?
- Do you have any non-financial competing interests in relation to this paper?

If you can answer no to all of the above, write 'I declare that I have no competing interests' below. If your reply is yes to any, please give details below.

I declare that I have no competing interests

I agree to the open peer review policy of the journal. I understand that my name will be included on my report to the authors and, if the manuscript is accepted for publication, my named report including any attachments I upload will be posted on the website along with the authors' responses. I agree for my report to be made available under an Open Access Creative Commons CC-BY license (http://creativecommons.org/licenses/by/4.0/). I understand that any comments which I do not wish to be included in my named report can be included as confidential comments to the editors, which will not be published.

I agree to the open peer review policy of the journal

To further support our reviewers, we have joined with Publons, where you can gain additional credit to further highlight your hard work (see: https://publons.com/journal/530/gigascience). On publication of this paper, your review will be automatically added to Publons, you can then choose whether or not to claim your Publons credit. I understand this statement.

Yes
